# Exploring DNA Topoisomerase I Ligand Space in Search of Novel Anticancer Agents

**DOI:** 10.1371/journal.pone.0025150

**Published:** 2011-09-22

**Authors:** Malgorzata N. Drwal, Keli Agama, Laurence P. G. Wakelin, Yves Pommier, Renate Griffith

**Affiliations:** 1 Department of Pharmacology, School of Medical Sciences, University of New South Wales, Sydney, Australia; 2 Laboratory of Molecular Pharmacology, Center for Cancer Research, National Cancer Institute, Bethesda, Maryland, United States of America; University of Helsinki, Finland

## Abstract

DNA topoisomerase I (Top1) is over-expressed in tumour cells and is an important target in cancer chemotherapy. It relaxes DNA torsional strain generated during DNA processing by introducing transient single-strand breaks and allowing the broken strand to rotate around the intermediate Top1 – DNA covalent complex. This complex can be trapped by a group of anticancer agents interacting with the DNA bases and the enzyme at the cleavage site, preventing further topoisomerase activity. Here we have identified novel Top1 inhibitors as potential anticancer agents by using a combination of structure- and ligand-based molecular modelling methods. Pharmacophore models have been developed based on the molecular characteristics of derivatives of the alkaloid camptothecin (CPT), which represent potent antitumour agents and the main group of Top1 inhibitors. The models generated were used for *in silico* screening of the National Cancer Institute (NCI, USA) compound database, leading to the identification of a set of structurally diverse molecules. The strategy is validated by the observation that amongst these molecules are several known Top1 inhibitors and agents cytotoxic against human tumour cell lines. The potential of the untested hits to inhibit Top1 activity was further evaluated by docking into the binding site of a Top1 – DNA complex, resulting in a selection of 10 compounds for biological testing. Limited by the compound availability, 7 compounds have been tested *in vitro* for their Top1 inhibitory activity, 5 of which display mild to moderate Top1 inhibition. A further compound, found by similarity search to the active compounds, also shows mild activity. Although the tested compounds display only low *in vitro* antitumour activity, our approach has been successful in the identification of structurally novel Top1 inhibitors worthy of further investigation as potential anticancer agents.

## Introduction

DNA topoisomerases relax DNA torsional strain generated during replication, transcription, recombination, repair, and chromosome condensation [1], and are therefore vital to all cells undergoing division. The relaxation of DNA supercoiling by topoisomerase I (Top1) is enabled by a mechanism of controlled rotation around a transient DNA single-strand break [2], [3]. During this process, the enzyme forms an intermediate covalent complex with the DNA, mediated by a bond between the active site tyrosine (Tyr723 in human Top1) and the cleaved phosphate group, as reviewed in [1]. At this stage, the enzyme is particularly vulnerable to a group of anticancer agents that reversibly trap the complex by intercalating between DNA base pairs at the cleavage site (“poisons”), thereby inhibiting religation [4]. Collision of the replication machinery with the trapped complex leads to irreversible DNA strand breaks [5], resulting in activation of apoptotic and cell cycle arrest pathways [6], [7].

**Figure 1:**
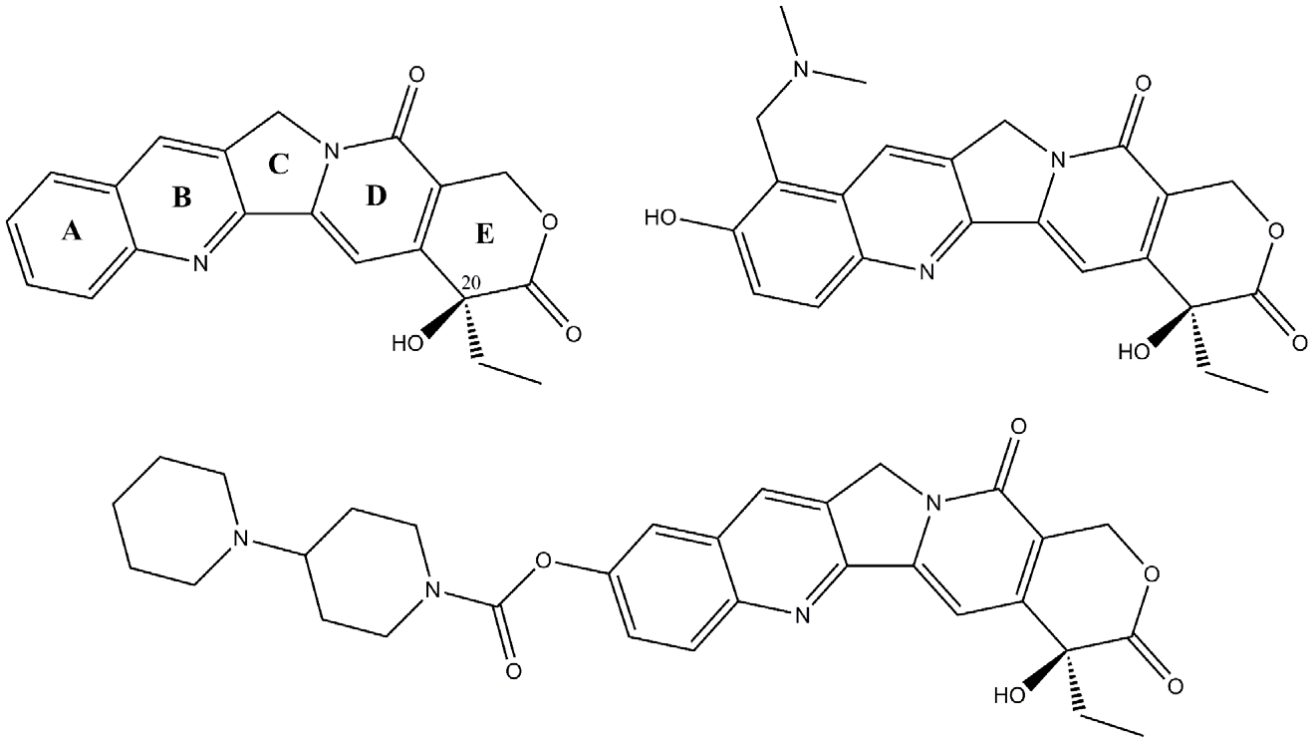
Chemical structures of camptothecins. Shown are the structures of camptothecin (CPT; top left), topotecan (TTC; top right), and irinotecan (CPT-11; bottom).

The main group of Top1 poisons are derivatives of the alkaloid camptothecin (CPT, Figure 1) isolated from the bark of the Chinese tree *Camptotheca accuminata* [8]. Although camptothecin was found to be clinically active, further development was hindered due to problems with solubility and severe side-effects [9], [10]. After identification of Top1 as the target of camptothecin [11], interest in the development of CPT derivatives as anticancer agents has increased. Today, two CPT analogues, topotecan and irinotecan (TTC and CPT-11, Figure 1) are used clinically for the therapy of both leukaemia and solid tumours [7]. However, their application is limited due to chemical instability of the hydroxylactone ring, multidrug-resistance and dose-limiting side-effects [12]–[14]. Due to the shortcomings of the camptothecins, there is much interest in the development of structurally different Top1 inhibitors. Homocamptothecins, containing a 7-membered lactone, and camptothecin derivatives with a 5-membered ketone ring have been developed to overcome the instability of the hydroxylactone ring [13], [15], [16]. Focus has also been put on the development of non-camptothecin Top1 inhibitors, such as indolocarbazoles, indenoisoquinolines and phenanthridines [12], [17]. Several compounds are currently under clinical investigation [17].

The use of pharmacophore models is a well-known approach in computer-aided drug design and its successes in the development of novel inhibitors have been reported [18]. In the absence of knowledge of the structure of the target, ligand-based pharmacophore models can be developed using activity data for a pool of ligands from an *in vitro* enzyme inhibition assay [19]. By contrast, structure-based molecular modelling methods, e.g. structure-based pharmacophores and docking, are an alternative approach when structural information about the target protein is available [20]. Here, the availability of several Top1 – DNA – drug crystal structures [4], [21]–[24] as well as topoisomerase I inhibition data [25], enabled the identification of structurally novel topoisomerase I inhibitors using a combination of structure- and ligand-based molecular modelling techniques. The success of our approach has been confirmed by the identification of 6 compounds with mild to moderate Top1 inhibitory activity.

## Results

### A new pharmacophore feature – cyclic π interaction

A crucial step in the development of high-quality pharmacophore models is the selection of the appropriate chemical features [26] enabling the complete description of the interactions between a ligand and its biological target. Stacking interactions between Top1 inhibitors and the DNA bases at the cleavage site have been reported to play an important role in the binding of the drug to the Top1 – DNA cleavable complex [27]. However, the ring aromatic feature present in Discovery Studio software (Accelrys, USA) which was used for the development of all pharmacophore models (see Methods), was observed not to map all rings capable of π-interactions, for example the DNA base thymine. This led us to the development of a new pharmacophore feature necessary for this project – the cyclic π-interaction feature (CYPI). We have designed this feature to map all five- and six-membered rings capable of π-interactions and have used it in the generation of the following pharmacophores.

### Ligand-based Top1 poison pharmacophores

**Figure 2:**
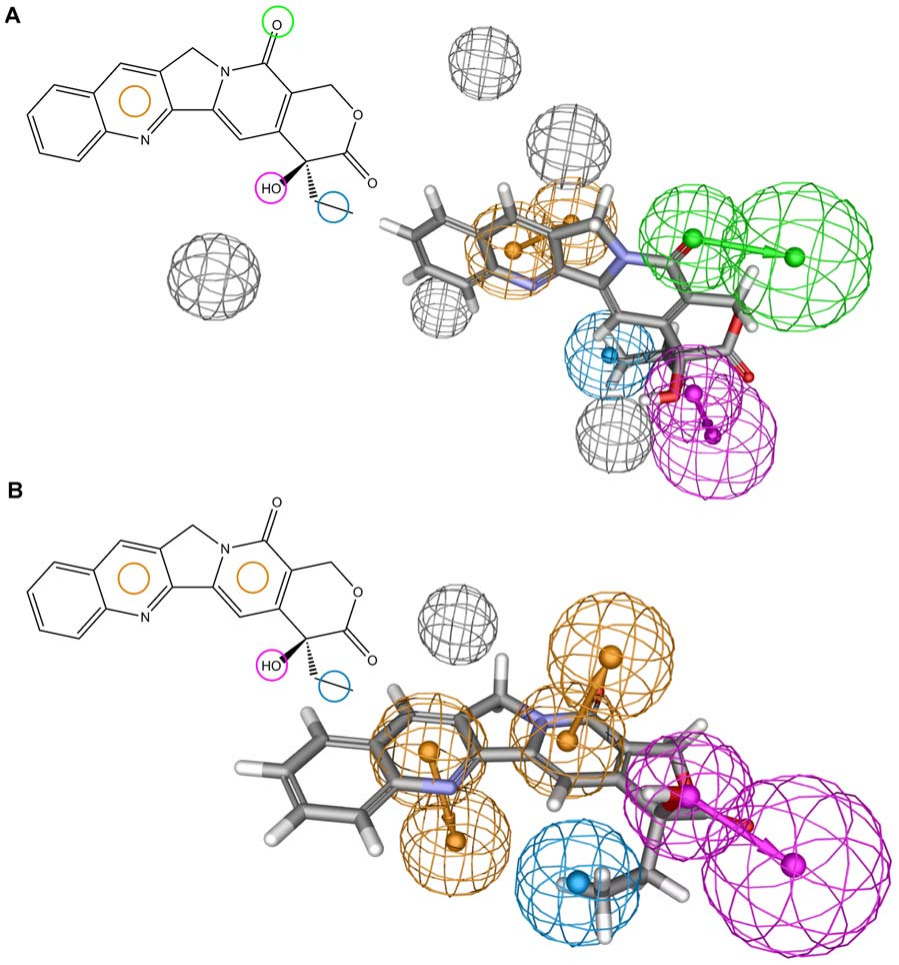
Ligand-based pharmacophores for camptothecin derivatives. 2D and 3D representations of the first (A) and second (B) pharmacophore hypothesis and their mapping to camptothecin. Cyclic π-interaction (CYPI) features are shown in orange, hydrogen bond acceptor (HBA) features in green, hydrogen bond donor (HBD) features in pink, hydrophobic (HYD) features in blue, and excluded volumes in gray. Mesh spheres in the 3D representations symbolize location constraints, with the second sphere for CYPI, HBA and HBD features showing the proposed location of the interacting atoms of the target (protein or DNA). Camptothecin in the 3D representation is shown in colour-coded sticks (carbon: gray, hydrogen: white, nitrogen: blue, oxygen: red).

The training set for the ligand-based pharmacophores was generated from camptothecin derivatives with known IC_50_ values measured in a Top1 poison specific assay [28]–[32]. Camptothecin derivatives are the only Top1 selective poisons with IC_50_ data available from a DNA cleavage assay. From the 77 compounds that have been tested, 27 were selected as a representative set (Table S1), chosen because of their structural diversity and activity spread. 3D QSAR pharmacophore hypotheses were generated as described in the Methods section. From the 10 hypotheses generated, two were selected based on statistical analysis. These two hypotheses show high correlation with biological activity (0.96 and 0.94, respectively) as well as high statistical significance (99%). The hypotheses also show similarities in the pharmacophore feature selection and placement (Figure 2). Both models place a hydrogen bond donor (HBD, pink) feature on the 20-OH group of camptothecin which is consistent with the importance of the stereochemistry at this position for compound activity. Furthermore, both pharmacophores contain a cyclic π-interaction (CYPI, orange) feature at the pyridine ring and a hydrophobic (HYD, blue) feature at the 20-ethyl group. Nevertheless, the hypotheses display three important differences – the placement of an HBA feature on the oxygen of the pyridine-2-one in hypothesis 1, the placement of a CYPI feature on the pyridine-2-one ring in hypothesis 2, as well as the placement of excluded volumes (gray). Thus, given the above characteristics, both pharmacophore hypotheses were kept for virtual screening.

### Structure-based Top1 poison pharmacophore

**Figure 3:**
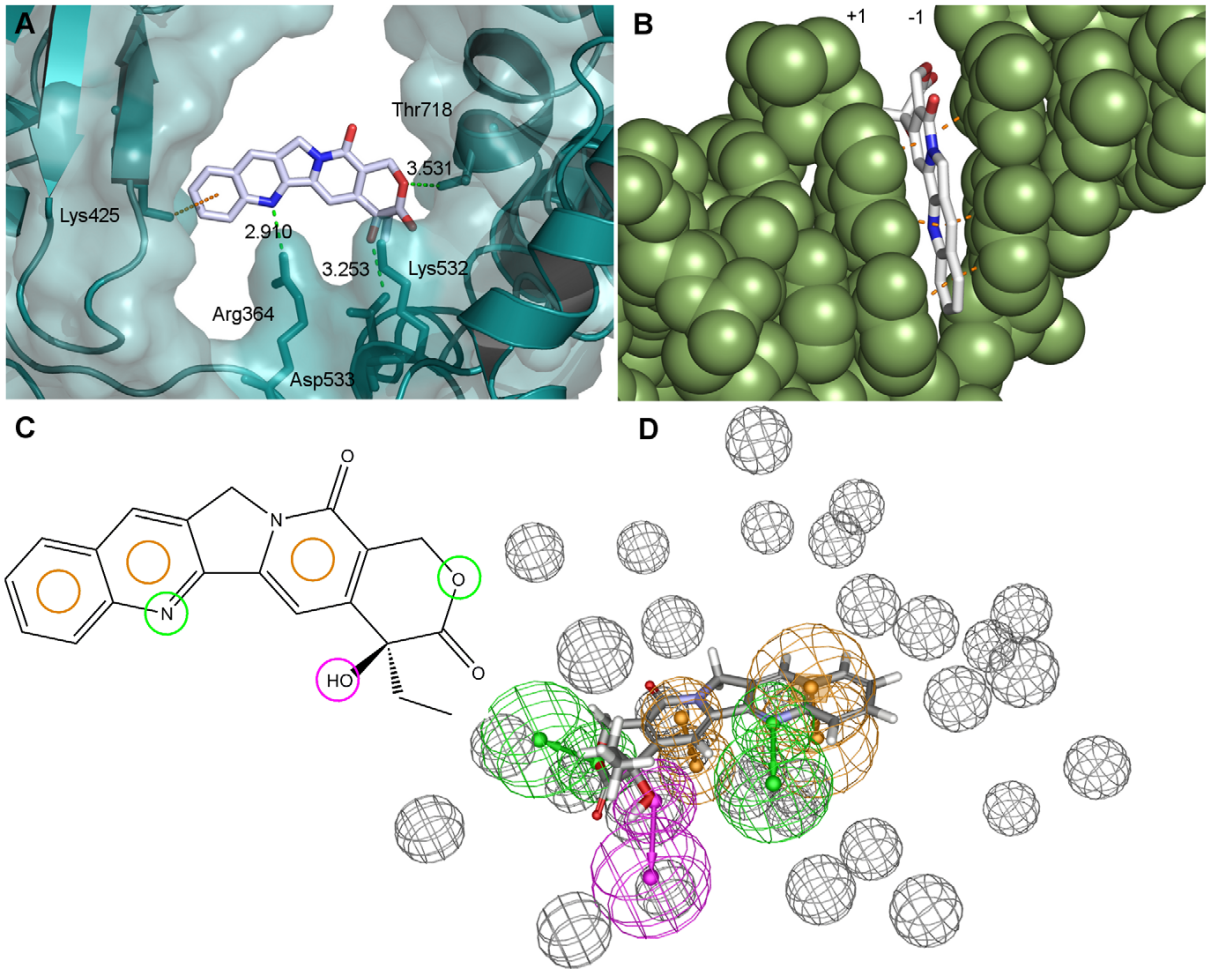
Structure-based pharmacophore development for camptothecins. (A) Camptothecin in the Top1 active site (from PDB file: 1T8I) viewed down the DNA helix axis. The protein backbone is shown as a solid ribbon and the protein surface in soft blue. Possible hydrogen bonds and π-interactions between camptothecin and Top1 are shown in green and orange, respectively (dotted lines), with distances between heavy atoms shown (Å). The amino acids involved are represented as sticks. (B) DNA - camptothecin interactions. DNA shown in green in space-filling mode. Examples of π-π interactions between camptothecin and flanking DNA bases are indicated in orange (dotted lines). (C) 2D representation of the structure-based pharmacophore for camptothecins and its mapping to CPT. The pharmacophore is an intersection between the camptothecin and the topotecan pharmacophores. (D) 3D representation of the pharmacophore used in database screening. Features and camptothecin colours are represented as in Figure 2.

Structure-based pharmacophore models can be generated when structural information of protein-ligand complexes is available. In the case of Top1, several crystal structures of the ternary enzyme-DNA-drug complex have been published [21]–[24]. Here, two of these crystal structures were selected for the development of structure-based Top1 poison pharmacophores. The selection was based on the fact that both structures contain similar drug molecules, camptothecin (PDB code: 1T8I [23]) and topotecan (PDB code: 1K4T [24]), and both binding sites are wild-type sequences. The protein-ligand and DNA-ligand interactions in the selected crystal structures were identified as described in the Methods section. In general, it was observed that protein-drug interactions are formed by hydrogen bonds and π-cation interactions (Figure 3A), whereas the drug contacts with the DNA are present in the form of π-π-interactions (Figure 3B). The pharmacophore features were placed according to interactions common to both crystal structures. The shape of the binding pocket was taken into account by adding excluded volumes. An additional excluded volume was placed to enable the distinction between active and inactive stereoisomers of camptothecin. The final pharmacophore model (Figure 3C) consists of three CYPI features, two hydrogen bond acceptors (HBA) and one hydrogen bond donor (HBD) feature. The positions of the CYPI and HBD features are similar to those of the ligand-based pharmacophores.

### Virtual database screening

The compound database of the National Cancer Institute (NCI, USA) contains a collection of about 240 000 compounds, many of which have been tested *in vitro* for cytotoxicity against human tumour cell lines. To retrieve novel Top1 inhibitors, and potential anticancer agents, the Top1 poison pharmacophores were applied sequentially in virtual screening of the NCI database (Table 1). Because the software and definition of the CYPI feature do not allow mapping to fused ring systems that share aromatic bonds, the two CYPI features of the structure-based pharmacophore placed on the quinoline rings of camptothecin (Figure 3C) had to be merged into one feature with a larger location constraint (Figure 3D), before application in database searching. Screening with the ligand-based and structure-based pharmacophores (without excluded volumes) retrieved a hit list of 3474 compounds, which was further reduced by applying a drug likeness filter based on Lipinski's rule of 5 [33]. The resulting list, called generation 1 hit list (see Table 1), consists of 1763 structurally diverse compounds. 2.7% of the compounds are camptothecin derivatives, and over half of them (29/46) are ranked within the top 100. As these compounds were not included in the training set for the pharmacophore generation, this represents a positive control for our methodology. Visual inspection of the generation 1 hit list, however, suggested that many molecules had been retrieved that might be too large for the binding pocket. Thus, a second screening was performed using a structure-based pharmacophore that contained excluded volumes to mimic the shape of the binding site. This screening reduced the number of hits to 756 compounds (generation 2 hit list, Table 1), 6.3% of which are camptothecin derivatives. In comparison with the generation 1 hit list, however, only a low percentage of the CPT derivatives were ranked within the top 100 compounds (2 out of 46). 21.8% of the generation 2 compounds have been tested against human tumour cell lines (publicly available NCI data), with growth inhibition of some cell lines reaching GI_50_ (cell growth inhibition by 50%) concentrations in the nano-molar range. Both the generation 1 and 2 hit lists were used for the selection of compounds for biological testing.

**Table 1:** Virtual screening of the NCI database.

National Cancer Institute (NCI, USA) database	about 240 000 compounds
Screening with ligand-based pharmacophores	7175 hits
Screening with structure-based pharmacophore (no excluded volumes)	3474 hits
Drug likeness filter	1763 hits^1^
Addition of excluded volumes to mimic shape of binding pocket	746 hits^2^

1 generation 1 hit list,

2 generation 2 hit list.

### Expert selection and molecular docking

The top 20 compounds of the generation 1 and 2 hit lists were inspected individually for further investigation. This first “expert selection” method selected according to the following criteria: (1) the compound is not a camptothecin derivative; (2) it has not been tested for Top1 inhibition; and (3) it is dissimilar to other compounds already selected for further investigation. Based on this method, 22 compounds were chosen and their possible fit into the Top1-DNA binding pocket was investigated using docking into the topotecan crystal structure (PDB code: 1K4T [24], see Methods).

**Figure 4:**
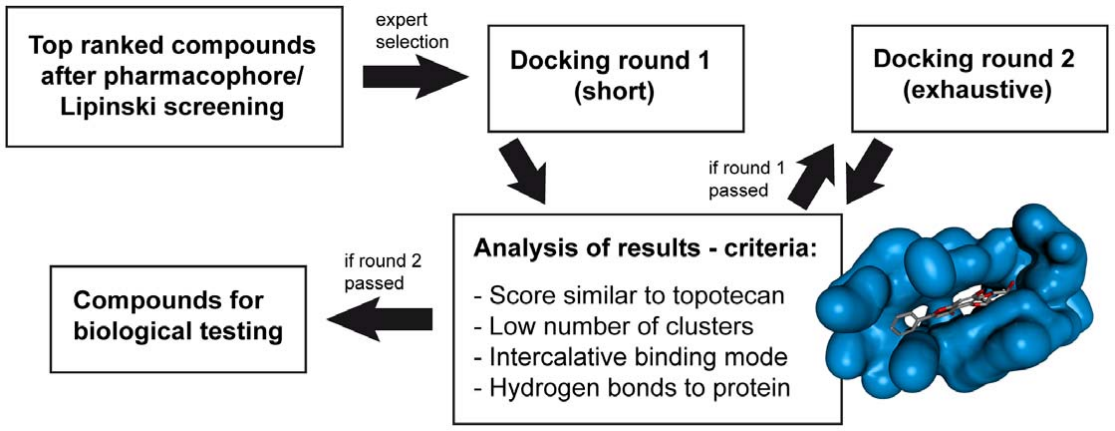
Selection of compounds for biological testing. Overview of the procedure used to select compounds for biological testing. See text for details.

**Figure 5:**
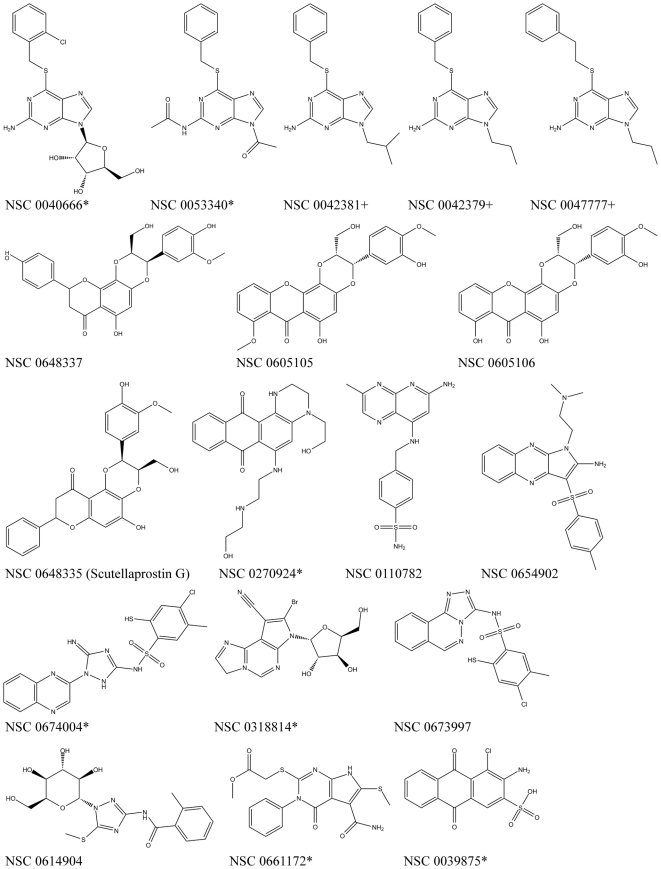
Chemical structures of compounds suggested for biological testing. Compounds available for testing and tested in a Top1 DNA cleavage assay are marked with an asterisk (*), if identified through virtual screening (Table 2), and with a cross (+), if identified through a similarity search.

As described above, X-ray structures of ternary complexes have revealed that the binding of known Top1 poisons is stabilised not only by stacking interactions with the DNA, but also by hydrogen bonds with the protein. Thus, the binding site defined in the docking simulations consisted of both DNA and protein residues close to the DNA cleavage site (Figure S1). All docking settings were tested beforehand in control dockings of topotecan back into its crystal structure, and the use of the optimised parameters resulted in docking poses with a root mean square deviation (RMSD) of 0.91 Å to the ligand position in the crystal structure. In control dockings, water molecules were found to have no significant effect on the docking scores and binding pose prediction, and were thus deleted from the binding site. The docking procedure consisted of an initial, short docking run, and an exhaustive run (Figure 4; see Methods section). If a compound showed satisfactory results in the short docking, it was passed to the second docking round. The analysis of docking results was based on the docking scores, as well as on the poses, the clusters of poses, and the interactions observed between the ligand and the binding pocket. In particular, in a second “expert selection”, a docking run was considered satisfactory, if (1) the docking score was similar to the scores obtained with control dockings of topotecan, (2) the number of clusters of docking poses was low, (3) the compound showed an intercalative binding mode between the DNA bases at the cleavage site, meaning that stacking interactions were detected and (4) the compound showed hydrogen bonds to the protein side chains of the binding pocket. In order to pass the first (short) docking round, the fulfilment of at least 3 criteria was required. This led to the elimination of 6 compounds (Table S2). In contrast, to pass the second (exhaustive) round of docking, and therefore to be considered for biological testing, all criteria were required to be satisfied. Based on this standard, 9 compounds were selected for biological testing (Table 2 and Figure 5). One additional compound (NSC 0040666) that showed a high number of docking clusters, otherwise fulfilled all remaining criteria, and was added to the test proposal list.

**Table 2:** Compounds that have passed into the second docking round.

List	Rank	Compound	Docking result (docking round 2)
*1*	3	NSC 0654902	All criteria fulfilled. Proposed for testing, but not available.
*1*	5	NSC 0648335	All criteria fulfilled. Proposed for testing, but not available.
*1*	7	NSC 0109617	Few interactions with protein. Not proposed for testing.
*1*	9	NSC 0614904	All criteria fulfilled. Proposed for testing, but not available.
*1*	12	NSC 0040666	Many possible binding modes, but other criteria fulfilled. Biol. testing.
*1*	15	NSC 0674004	All criteria fulfilled. Biol. testing.
*1*	16	NSC 0270924	All criteria fulfilled. Biol. testing.
*1*	17	NSC 0149871	Few interactions with protein. Not proposed for testing.
*1*	18	NSC 0648201	Few interactions with protein. Not proposed for testing.
*1*	19	NSC 0332448	Either intercal. mode or interactions with protein. Not proposed for testing.
*2*	13	NSC 0295494	Moderate docking scores. Not proposed for testing.
*2*	15	NSC 0661172	All criteria fulfilled. Biol. testing.
*2*	17	NSC 0318814	All criteria fulfilled. Biol. testing.
*2*	18	NSC 0039875	All criteria fulfilled. Biol. testing.
*2*	19	NSC 0053340	All criteria fulfilled. Biol. testing.
*2*	20	NSC 0090917	Low scores. Not proposed for testing.

### Biological testing of promising compounds

**Figure 6:**
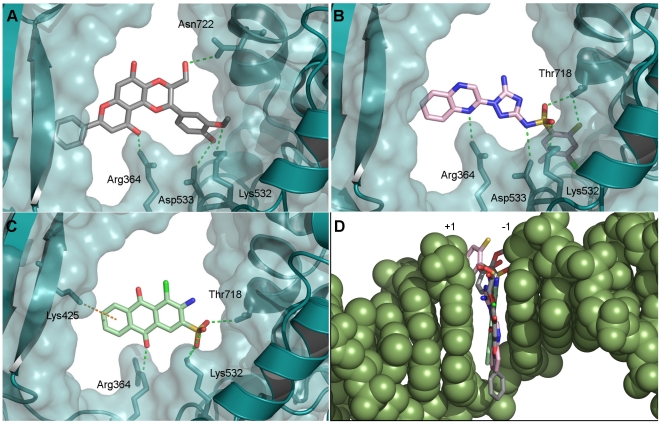
Docking results. Interactions between protein side chains and docked ligand: (A) Scutellaprostin G, (B) NSC 0674004, (C) NSC 0039875. (D) Overlay of docking poses of Scutellaprostin G (gray carbons), NSC 0674004 (pink carbons) and NSC 0039875 (green carbons) within the DNA cleavage site. See Figure 3 for details of representation and colours.

**Figure 7:**
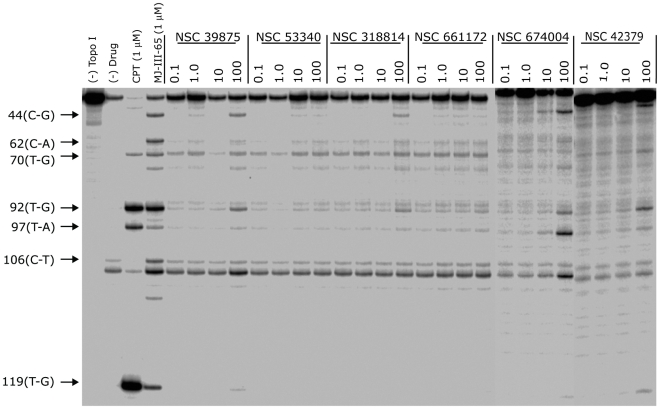
Top1-mediated DNA cleavage induced by tested compounds. (lane 1) DNA alone; (lane 2) Top1 alone; (lane 3) camptothecin, 1 µM; (lane 4) MJ-III-65, 1 µM; (lanes 5–28) Top1 + NCI compounds indicated at 0.1, 1, 10 and 100 µM concentrations, respectively. The numbers on the left and arrows indicate cleavage site positions.

The Top1 DNA cleavage assay is a Top1 poison-specific method [34], [35], which was used to assess the biological function of the 10 compounds selected from *in silico* screening. However, out of 10 compounds only seven were available for testing, with three and four compounds from the generation 1 and 2 hit lists, respectively. One particular compound, Scutellaprostin G (NSC 0648335, Figure 5), emerged as the most promising compound of the virtual screening. This flavonoid isolated from the plant *Scutellaria prostata* [36] is highly ranked in both hit lists, receives high docking scores (similar to topotecan), shows many interactions with the binding site (Figure 6A and D), and displays promising GI_50_ values in the low micromolar range (publicly available NCI data). Unfortunately, Scutellaprostin G was not available for testing, which prompted us to perform a similarity search within the NCI database (see Methods section; Tanimoto similarity [37] >92%), taking into account presence in the hit lists and satisfactory docking results. However, none of the identified compounds were available for testing, either. The Top1 inhibitory activity of the available 7 compounds (Table 2) was assessed semi-quantitatively, by comparing their activity to the activity of 1 µM camptothecin (CPT) [34], [35]. The results of the assay are shown in Figure 7 and summarized in Table 3. From the generation 1 compounds, a 2-mercaptobenzenesulphonamide derivative (NSC 674004; rank 16 in generation 1 hit list) shows activity classified as +/++ (25–75% of CPT activity). In addition, three compounds of the generation 2 hit list (NSC 0661172, NSC 0318814, and NSC 0053340; ranks in generation 2 hit list: 15, 17, and 19, respectively) show + activity (25–50% CPT activity), and one compound (NSC 0039875, rank 18 in generation 2 hit list) displays +/++ Top1 inhibitory activity.

**Table 3:** Results of Top1 DNA cleavage assay and cytotoxicity assay.

List	Compound	CAS-RN^1^	Top1 inhibition^2^	Cytotoxic activity: GI_50_ 3
*1*	NSC 0674004	185216-64-2	+/++	Between 15.8 and 100 µM^4^
*2*	NSC 0661172	153621-30-8	+	>10 µM for all cells^4^
2	NSC 0318814	76867-10-2	+	Between 20.0 and >100 µM^5^
2	NSC 0053340	93009-81-5	+	Between 23.4 and >100 µM^5^
2	NSC 0039875	736072-20-1	+/++	>100 µM for all cells^5^
*Simil.*	NSC 0042379	92556-40-6	+	Between 20.0 and >100 µM^5^
*Control*	Camptothecin(CPT, NSC 0094600)	7689-03-4	++++	Between 10 nM and 1.3 µM^4^

1 Chemical Abstracts Registration Number.

2 Top1 inhibition ranking: 0 (no activity); + (20–50% of 1 µM CPT activity); ++ (50–75% of 1 µM CPT activity); +++ (75–100% of 1 µM CPT activity); ++++ (equipotent or more potent than 1 µM CPT).

3 Cytotoxic activity measured in the US National Cancer Institute (NCI) 60 human tumour cell line anticancer drug screen [47]. GI_50_ corresponds to the concentration of the drug (molar) resulting in a 50% growth inhibition.

4 determined previously, published on-line (http://dtp.nci.nih.gov/); in case of camptothecin, the data has been averaged from six experiments, for all other compounds, only one experiment has been performed.

5 determined during this work, data from single experiment.

As an additional test, the 5 compounds with confirmed Top1 inhibitory activity have also been tested for cytotoxicity against the NCI panel of 60 human tumour cell lines (Table 3). Two of the hit compounds (NSC 0674004 and 0661172), and camptothecin, had been tested for antitumour activity previously and the results had been published on-line at the NCI database website (see Methods). Preliminary testing of the remaining compounds has been performed during this work. The cytotoxic activity is expressed as cell growth inhibition (GI_50_) after 48 h incubation with the drug. As summarized in Table 3, three of the tested compounds (NSC 0674004, 0318814, and 0053340) have GI_50_ values within the range 15 to 25 µM for some cell lines. The highest cytotoxic activity was observed in renal cancer (all three compounds), breast cancer (NSC 0053340 and 0318814), and non-small cell lung cancer (NSC 0674004) cell lines. The remaining two compounds (NSC 0661172 and 0039875) show GI_50_ values greater than 10 or 100 µM, respectively.

### Similarity search within NCI hit lists

Based on the five active compounds identified in the Top1 cleavage assay, we performed another similarity search within the NCI database (as described above), with the objective of obtaining compounds with similar structures, but potentially higher activity. This search led to the identification of five compounds (Figure 5) present in the virtual screening hit lists, all of which display satisfactory results in exhaustive docking runs (as described above). However, only three of these compounds were available for testing, each being a purine derivative with higher docking scores, but lower pharmacophore rankings than NSC 0053340. Of these three compounds, only one, NSC 0042379, shows activity in the Top1 DNA cleavage assay (Table 3), with a potency and cytotoxicity similar to NSC 0053340.

### Docking poses of most active compounds

The docking poses of the two most active compounds, NSC 0674004 and NSC 0039875, were analysed and compared to the docking pose of Scutellaprostin G (Figure 6, views down the DNA axis in A to C, and from the major groove in D). The poses shown were obtained from exhaustive docking runs. In particular, each pose represents not only the best-scored pose of the entire docking run for this ligand, but also the best-scored pose of the biggest cluster of solutions (clustering: 2.0 Å), and can thus be regarded as a representative putative binding mode. Analysis and superimposition of the docking poses reveals that the three compounds intercalate between the DNA base pairs at the cleavage site, each providing three aromatic rings for extensive base pair stacking, and form hydrogen bonds to the side chains of the Top1 residues Arg364 and Lys532. Furthermore, Scutellaprostin G and NSC 0674004 display an additional hydrogen bond to Asp533, and NSC 0039875 forms a π-cation interaction with Lys425. This intercalative binding mode, and these Top1 side chain interactions, are common to the camptothecin and topotecan ternary complexes, and this similarity encourages confidence in the reliability of the docking.

## Discussion

We report the development of ligand- and structure-based topoisomerase I inhibitor pharmacophore models and their application in virtual database screening for the identification of structurally novel inhibitors. To limit the number of hits to be tested, our methods were extended to include the use of a drug-likeness filter, molecular docking, and expert selection of compounds. To our knowledge, this study represents the first use of combined pharmacophore modelling and docking techniques in the topoisomerase I poison field. This combination of methods has enabled us to select 15, and test 10 molecules from the 240,000-compound National Cancer Institute (NCI) database, 6 of which show topoisomerase I inhibitory activity. This represents a hit rate of at least 60%, given that a number of highly rated compounds, for example Scutellaprostin G, were not available for testing. A similar hit rate has recently been obtained by Dong and colleagues [38], who used the crystal structure of the camptothecin-Top1-DNA complex for docking-based *in silico* screening of the commercial SPECS database. These high hit rates compare very favourably with those of high-throughput screening assays [39], and highlight the value of computational methods and knowledge-based selection in the drug development process. Moreover, our findings support the notion that a combination of ligand- and structure-based molecular modelling methods, and therefore the use of all available knowledge, might be the best strategy for a successful computer-aided drug design [40], [41].

Despite the success of our methodology in identifying compounds with topoisomerase I inhibitory ability, their cytotoxic potency is only modest, and does not correlate with the Top1 inhibitory activity (see Table 3). As disappointing as it might be, this is perhaps not surprising. The prediction of cytotoxic compounds was not the focus of our study, but an additional test towards the development of new anticancer agents targeting Top1. Here, we have not attempted to model cytotoxicity, since cytotoxic activity of a compound is a complex property that depends on many factors such as drug solubility, cellular uptake, stability, selectivity, off-target activity, or resistance, which makes its prediction very difficult. Lipinski's rule of five was applied during this study to reduce the number of hits and to focus the hit list towards potentially orally available drugs. However, it has been proposed that the chemical space occupied by anticancer drugs is not a subset of the drug-like compound space, as defined by Lipinski's rule, but is of much greater volume [42]. This implies that in order to focus a compound library towards anticancer agents, it might be necessary to apply other molecular filters that have been trained on specific groups of anticancer drugs. To our knowledge, no such filter has been developed for Top1 inhibitors yet: an objective that might present an interesting future direction for use of our pharmacophores.

A noticeable observation during our work was that neither the ranking after the pharmacophore screening, nor the docking scores correlate with the actual Top1 inhibitory activity. The scoring problem is a known challenge in pharmacophore modelling as well as docking approaches [40]. The ranking of compounds retrieved by a pharmacophore screening is only based on a geometrical fit of features and the relative energy of the fitted conformation, and does not necessarily correlate with the actual binding affinity or inhibitory activity. Low structural diversity of the training set during the development of ligand-based pharmacophores limits the predictability of the generated pharmacophores. Structure-based pharmacophores, by contrast, do not include any quantitative information about binding affinities, which restricts their use to a hit list filtering function. Moreover, comparative studies have shown that the correlation between docking scores and *in vitro* activities is generally low [43] and that the results are target-dependent [40]. Despite this, the GOLD program has been shown to perform well in the prediction of binding poses in protein targets [43]. Since GOLD has not been validated for docking into DNA, we have tested different settings and scoring functions in control dockings back into the original crystal structures, and observed that the GOLD scoring function gave the best results in terms of RMSD to the original ligand position. Similar results were obtained by Dong and colleagues [38] who also tested other docking programs, leading to the conclusion that GOLD and its scoring function are suitable for this molecular target. Although the compound ranking according to the pharmacophore fitting and the docking scores leaves room for improvement, it is important to emphasize that our selection of compounds was not only based on these two values, but included a visual inspection of the docking poses, and that this combined approach has proven successful, as it resulted in the identification of active compounds.

Although none of our tested compounds displays high cytotoxic activity in human tumour cell lines, our study has identified several different chemical scaffolds that might be worth further investigation in the Top1 inhibition and anticancer field. One of the most promising scaffolds derives from 9,10-dioxoanthracene-2-sulfonic acid, with NSC 0039875 showing comparatively high Top1 inhibitory ability, albeit little cytotoxicity (Table 3). However, this does not necessarily imply inactivity of other members of the chemical family, since one of the hits identified in a structure-based virtual screening study of Dong *et al.* [38] has the same scaffold as NSC 0039875. The hit compound , 4-(p-tolylsulfonyl)-naphtho-[2,3-g][2], [1], [3]benzothiadiazole-6,11-dione (**38_1**), is a Top1 inhibitor and shows *in vitro* antitumour activity in the A-549 non-small-cell lung cancer cell line (IC_50_ = 0.50±0.0021 µM), as well as the LOVO (colon cancer, IC_50_ = 43±1.1 µM) and MDA-MB-435 (breast cancer, IC_50_ = 25±0.97 µM) cell lines [38]. The structural differences between the two compounds include: the substitution of a toluene group for a hydroxyl group attached to the sulphur atom of NSC 0039875, the substitution of a thiazole ring attached to the third ring of the compound for chlorine, and the presence of an amino substituent in NSC 0039875 (Figure 5). It should be noted that the binding mode predicted for NSC 0039875 differs from the pose of the structurally related compound **38_1** (see above) regarding the orientation within the binding pocket. Whereas the sulphate group of NSC 0039875 forms hydrogen bonds to Lys532 and Thr718 (Figure 6C), the sulphate group of **38_1** points into the opposite direction. In both cases, however, the major axes of the molecular scaffolds lie parallel to the DNA bases at the cleavage site. To investigate whether the differences in the binding modes are due to the use of two distinct crystal structures and slightly different docking parameters, or whether the differences result from chemical variations between the compounds, we performed a short control docking of compound **38_1** using our settings. The best-scored pose showed a similar orientation to the one described by Dong and colleagues [38]. When analysing the other poses of the docking run, however, we found that the largest cluster of poses (RMSD clustering: 2.0 Å) showed an orientation similar to the one observed for NSC 0039875, suggesting that both orientations of the scaffold in the binding pocket are plausible. Irrespective of the binding mode of these compounds, derivatives of 9,10-dioxoanthracene-2-sulfonic acid represent an interesting chemical scaffold for systematic QSAR studies.

Other chemical scaffolds worthy of further investigation include purine derivatives such as NSC 0053340 and NSC 0042379, pyrimidine derivatives such as NSC 0318814, and analogues of 2-mercaptobenzenesulphonamide such as NSC 0674004, which all display activity in the Top1 inhibition assay and similarly high *in vitro* antitumour activity, with the highest potency in renal cancer cells (GI_50_ values between 15 and 25 µM, see Table 3). To our knowledge, none of these compounds have previously been tested for Top1 inhibition. In addition, although the activity of Scutellaprostin G has never been tested, we believe that this molecule and its analogues are a promising group of potential Top1 inhibitors. This is not only supported by the observations of good pharmacophore mappings and docking poses for Scutellaprostins, but also by the fact that Silibinin and other flavonoids structurally similar to Scutellaprostins have been identified as DNA intercalators and Top1 poisons [44]. Furthermore, four Scutellaprostins have been tested for cytotoxicity against the NCI 60 tumour cell line panel (Scutellaprostin A, B, D, and G), with GI_50_ concentrations located in the low micromolar range (publicly available NCI data). A comparison of the docking poses of the newly identified Top1 inhibitors, and Scutellaprostin G, revealed that all compounds manifest a similar intercalative binding mode between the DNA bases at the cleavage site (Figure 6D). Although comprising different scaffolds, all compounds possess an aromatic core typical for Top1 inhibitors. Moreover, as shown in Figure 6, the most active Top1 inhibitors identified in this study, NSC 0674004 and 0039875, as well as Scutellaprostin, display interactions with Top1 side-chains involved not only in the binding of camptothecins [21], [23], [24], but also indenoisoquinolines and indolocarbazoles [22], [23], suggesting a similar mechanism of action for all compounds.

In conclusion, the combination of pharmacophores, docking methods and expert assessment can be successfully applied in virtual database screening to retrieve known Top1 inhibitors, compounds with anticancer activity, as well as structurally new compounds with Top1 inhibitory activity. The hit compounds identified in this study, despite their low cytotoxic activity, can be regarded as promising starting points for future developments of anticancer drugs.

## Materials and Methods

### Ligand-based pharmacophores

Ligand-based pharmacophore models were generated using the Discovery Studio 2.5.5 package (Accelrys Software Inc. USA). A training set of 27 camptothecin derivatives was selected from the literature based on structural and functional diversity [28]–[32]. All compounds were sketched manually, their geometry was cleaned and their conformations were generated using the “best” option. Pharmacophore models were generated using the HypoGen [45] and HypoRefine algorithms. Uncertainty values were set to 2.0 and the following pharmacophore features were used: hydrogen bond acceptor (HBA), hydrogen bond donor (HBD), hydrophobic (HYD), cyclic π-interaction (CYPI). The CYPI feature is a user-defined feature that, in contrast to the ring aromatic feature present in Discovery Studio, maps all five- and six-membered rings capable of π-interactions. The definition of the feature was based on the ring aromatic feature present in the software, and extended by adding all fragments to be mapped. No restrictions were applied to the number of the pharmacophore features. A maximum of 5 excluded volumes was allowed. For the validation of pharmacophore hypotheses, Fischer randomization was used.

### Structure-based pharmacophores

Structure-based pharmacophores were developed based on the crystal structures of ternary DNA topoisomerase I-DNA-drug complexes. In particular, crystal structures of the drugs camptothecin and topotecan were used (PDB codes 1T8I [23] and 1K4T [24], respectively). Protein-ligand and DNA-ligand interactions were identified using the Discovery Studio Monitor function and visual inspection. Pharmacophore features were manually placed according to identified interactions. For the CYPI feature placement, the feature mapping protocol was used. A common pharmacophore was generated containing the features present in both crystal structures and an average of the features was calculated. Excluded volumes were placed to mimic the shape of the binding pocket using a Discovery Studio script. An additional excluded volume was manually placed to account for the lower activity of the 20-R stereoisomer (see Figure 1).

### Virtual database screening

The compound database of the National Cancer Institute (NCI2000), imported into Discovery Studio, was screened using the 3D Database Search protocol in the same software. The screening was performed sequentially. The hit list was filtered using Lipinski's rule of 5 [33].

### Docking

All dockings were performed with the program GOLD [46] version 4.1 and 5.0 using the crystal structure of the topotecan – Top1 – DNA complex (PDB code: 1K4T). Water molecules and ligands were deleted, hydrogens were added. The SH-group at the DNA cleavage site was mutated to OH. The binding site was defined as the cavity detected 7.5 Å around the initial ligand position. Flexible side chains were defined according to the residues observed to interact with the original ligand (Asn352, Glu356, Arg364, Lys425, Lys532, Asp533, and Thr718). For short runs, default settings were used. For exhaustive docking runs, the number of runs per ligand was increased from 10 to 100, the population size was increased to 1000, and the number of genetic algorithm operations was increased to 10^6^. Early termination was allowed if the 5 best poses were within 1.5 Å RMSD (only for exhaustive runs). The GoldScore function was used to rank the results. The docking poses were clustered based on 2.0 Å RMSD of heavy atoms.

### DNA cleavage assay

The Top1 inhibitory activity was measured in a DNA cleavage assay as described previously [25]. Briefly, 3′-radiolabeled DNA substrates are incubated with the Top1 enzyme and the drug to be tested, allowing the formation of ternary enzyme-DNA-drug complexes. The use of a strong protein denaturant, sodium dodecyl sulphate (SDS), leads to a denaturation of Top1 covalently bound to DNA, and the use of polyacrylamide gel electrophoresis enables the visualisation of cleavage products. The activity of a drug is measured semi-quantitatively, by comparison to the activity of 1 µM camptothecin (CPT). The scoring of the activity is defined as follows: 0: no activity; +: 25–50% CPT activity; ++: 50–75% CPT activity; +/++: 25–75% CPT activity; +++: 75–100% CPT activity; ++++: compound is equipotent or more potent than CPT.

### Working with the NCI2000 database

The virtual screening hits were analysed using the NCI2000 database websites (http://129.43.27.140/ncidb2/; http://dtp.nci.nih.gov/dtpstandard/dwindex/index.jsp). These websites contain information available for all compounds, including name, chemical structure, and cancer screening data. They can also be used to search for compounds based on Tanimoto similarity.

### Cytotoxicity assay

The cytotoxicity of selected compounds was measured in the NCI 60 human tumour cell line anticancer drug screen according to previously described protocols (see reference [47] and online at http://dtp.nci.nih.gov/branches/btb/ivclsp.html). Briefly, the cells were incubated with the drug for 48 h, and stained with sulforhodamine B. The absorbance was read from an automated plate reader at a wavelength of 515 nm, and the concentration of drug needed to inhibit cell growth by 50% recorded as a GI_50_ value. Except for the control compound camptothecin, the dose-response curves for the 60 cell lines were obtained from a single experiment. This was due to the low cytotoxic activity of the compounds.

## Supporting Information

Figure S1 **Binding site for the docking simulations.** The binding site was defined from the position of the ligand, camptothecin, in the crystal structure ternary complex with a DNA fragment and the top1 enzyme (PDB code 1T8I [23]) and includes both DNA (green) and protein (aqua) residues.
(TIF)

Table S1 **Training set for ligand-based pharmacophore generation.** 2D structures, IC_50_ values (in µM) for the inhibition of Top1, and references for molecules used in pharmacophore generation are given.
(DOCX)

Table S2 **Compounds that did not pass the first docking round.** Details on the hit list, the rank, the NSC code are given on compounds failing the first round of docking, as well as reasons for failure.
(DOCX)

## References

[1] Wang JC (2002). Cellular roles of DNA topoisomerases: a molecular perspective.. Nat Rev Mol Cell Biol.

[2] Stewart L, Redinbo MR, Qiu X, Hol WG, Champoux JJ (1998). A model for the mechanism of human topoisomerase I.. Science.

[3] Koster DA, Croquette V, Dekker C, Shuman S, Dekker NH (2005). Friction and torque govern the relaxation of DNA supercoils by eukaryotic topoisomerase IB.. Nature.

[4] Marchand C, Antony S, Kohn KW, Cushman M, Ioanoviciu A (2006). A novel norindenoisoquinoline structure reveals a common interfacial inhibitor paradigm for ternary trapping of the topoisomerase I-DNA covalent complex.. Mol Cancer Ther.

[5] Strumberg D, Pilon AA, Smith M, Hickey R, Malkas L (2000). Conversion of Topoisomerase I Cleavage Complexes on the Leading Strand of Ribosomal DNA into 5′-Phosphorylated DNA Double-Strand Breaks by Replication Runoff.. Mol Cell Biol.

[6] Zhang XW, Bin Xu CQ (1999). Apoptosis induction and cell cycle perturbation in human hepatoma Hep G2 cells by 10-hydroxycamptothecin.. Anti-Cancer Drugs.

[7] Pommier Y (2006). Topoisomerase I inhibitors: camptothecins and beyond.. Nat Rev Cancer.

[8] Wall ME, Wani MC, Cook CE, Palmer KH, Mcphail AT (1966). Plant Antitumor Agents. I. Isolation and Structure of Camptothecin a Novel Alkaloidal Leukemia and Tumor Inhibitor from Camptotheca Acuminata.. J Am Chem Soc.

[9] Gottlieb JA, Guarino AM, Call JB, Oliverio VT, Block JB (1970). Preliminary pharmacologic and clinical evaluation of camptothecin sodium (NSC-100880).. Cancer Chemother Rep.

[10] Creaven PJ, Allen LM (1973). Renal clearance of camptothecin (NSC-100880): effect of urine volume.. Cancer Chemother Rep.

[11] Hsiang YH, Hertzberg R, Hecht S, Liu LF (1985). Camptothecin induces protein-linked DNA breaks via mammalian DNA topoisomerase I.. J Biol Chem.

[12] Meng LH, Liao ZY, Pommier Y (2003). Non-camptothecin DNA topoisomerase I inhibitors in cancer therapy.. Curr Top Med Chem.

[13] Pommier Y (2009). DNA topoisomerase I inhibitors: chemistry, biology, and interfacial inhibition.. Chem Rev.

[14] Brangi M, Litman T, Ciotti M, Nishiyama K, Kohlhagen G (1999). Camptothecin Resistance.. Cancer Research.

[15] Bailly C (2003). Homocamptothecins: potent topoisomerase I inhibitors and promising anticancer drugs.. Crit Rev Oncol Hemat.

[16] Lansiaux A, Léonce S, Kraus-Berthier L, Bal-Mahieu C, Mazinghien R (2007). Novel Stable Camptothecin Derivatives Replacing the E-Ring Lactone by a Ketone Function Are Potent Inhibitors of Topoisomerase I and Promising Antitumor Drugs.. Mol Pharmacol.

[17] Pommier Y, Cushman M (2009). The indenoisoquinoline noncamptothecin topoisomerase I inhibitors: update and perspectives.. Mol Cancer Ther.

[18] Gao Q, Yang L, Zhu Y (2010). Pharmacophore based drug design approach as a practical process in drug discovery.. Curr Comput Aided Drug Des.

[19] Yang SY (2010). Pharmacophore modeling and applications in drug discovery: challenges and recent advances.. Drug Discov Today.

[20] Andricopulo AD, Salum LB, Abraham DJ (2009). Structure-based drug design strategies in medicinal chemistry.. Curr Top Med Chem.

[21] Chrencik JE, Staker BL, Burgin AB, Pourquier P, Pommier Y (2004). Mechanisms of camptothecin resistance by human topoisomerase I mutations.. J Mol Biol.

[22] Ioanoviciu A, Antony S, Pommier Y, Staker BL, Stewart L (2005). Synthesis and mechanism of action studies of a series of norindenoisoquinoline topoisomerase I poisons reveal an inhibitor with a flipped orientation in the ternary DNA-enzyme-inhibitor complex as determined by X-ray crystallographic analysis.. J Med Chem.

[23] Staker BL, Feese MD, Cushman M, Pommier Y, Zembower D (2005). Structures of three classes of anticancer agents bound to the human topoisomerase I-DNA covalent complex.. J Med Chem.

[24] Staker BL, Hjerrild K, Feese MD, Behnke CA, Burgin AB (2002). The mechanism of topoisomerase I poisoning by a camptothecin analog.. Proc Natl Acad Sci U S A.

[25] Dexheimer TS, Pommier Y (2008). DNA cleavage assay for the identification of topoisomerase I inhibitors.. Nat Protoc.

[26] Wolber G, Seidel T, Bendix F, Langer T (2008). Molecule-pharmacophore superpositioning and pattern matching in computational drug design.. Drug Discov Today.

[27] Song Y, Cushman M (2008). The Binding Orientation of a Norindenoisoquinoline in the Topoisomerase I–DNA Cleavage Complex Is Primarily Governed by π–π Stacking Interactions.. Journal of Physical Chemistry B.

[28] Lackey K, Besterman JM, Fletcher W, Leitner P, Morton B (1995). Rigid analogs of camptothecin as DNA topoisomerase I inhibitors.. J Med Chem.

[29] Lackey K, Sternbach DD, Croom DK, Emerson DL, Evans MG (1996). Water soluble inhibitors of topoisomerase I: quaternary salt derivatives of camptothecin.. J Med Chem.

[30] Luzzio MJ, Besterman JM, Emerson DL, Evans MG, Lackey K (1995). Synthesis and antitumor activity of novel water soluble derivatives of camptothecin as specific inhibitors of topoisomerase I.. J Med Chem.

[31] Uehling DE, Nanthakumar SS, Croom D, Emerson DL, Leitner PP (1995). Synthesis, topoisomerase I inhibitory activity, and *in vivo* evaluation of 11-azacamptothecin analogs.. J Med Chem.

[32] Wall ME, Wani MC, Nicholas AW, Manikumar G, Tele C (1993). Plant antitumor agents. 30. Synthesis and structure activity of novel camptothecin analogs.. J Med Chem.

[33] Lipinski CA, Lombardo F, Dominy BW, Feeney PJ (1997). Experimental and computational approaches to estimate solubility and permeability in drug discovery and development settings.. Adv Drug Deliv Rev.

[34] Kiselev E, Dexheimer TS, Pommier Y, Cushman M (2010). Design, synthesis, and evaluation of dibenzo[c,h][1,6]naphthyridines as topoisomerase I inhibitors and potential anticancer agents.. J Med Chem.

[35] Song YL, Shao Z, Dexheimer TS, Scher ES, Pommier Y (2010). Structure-Based Design, Synthesis, and Biological Studies of New Anticancer Norindenoisoquinoline Topoisomerase I Inhibitors.. J Med Chem.

[36] Kikuchi Y, Miyaichi Y, Tomimori T (1991). Studies on Nepalese Crude Drugs .14. New Flavonoids from the Root of Scutellaria-Prostrata Jacq Ex Benth.. Chemical & Pharmaceutical Bulletin.

[37] Willett P, Winterman V (1986). A Comparison of Some Measures for the Determination of Inter-Molecular Structural Similarity Measures of Inter-Molecular Structural Similarity.. Quantitative Structure-Activity Relationships.

[38] Dong G, Sheng C, Wang S, Miao Z, Yao J (2010). Selection of Evodiamine as a Novel Topoisomerase I Inhibitor by Structure-Based Virtual Screening and Hit Optimization of Evodiamine Derivatives as Antitumor Agents.. J Med Chem.

[39] Oprea T (2002). Current trends in lead discovery: Are we looking for the appropriate properties?. J Comput Aided Mol Des.

[40] Hein M, Zilian D, Sotriffer CA (2010). Docking compared to 3D-pharmacophores: the scoring function challenge.. Drug Discov Today.

[41] Talevi A, Gavernet L, Bruno-Blanch LE (2009). Combined Virtual Screening Strategies.. Curr Comput Aided Drug Des.

[42] Lloyd DG, Golfis G, Knox AJS, Fayne D, Meegan MJ (2006). Oncology exploration: charting cancer medicinal chemistry space.. Drug Discov Today.

[43] Plewczynski D, Łaźniewski M, Augustyniak R, Ginalski K (2011). Can we trust docking results? Evaluation of seven commonly used programs on PDBbind database.. J Comput Chem.

[44] Webb MR, Ebeler SE (2004). Comparative analysis of topoisomerase IB inhibition and DNA intercalation by flavonoids and similar compounds: structural determinates of activity.. Biochem J.

[45] Li H, Sutter J, Hoffmann R, Guener OF (2000). HypoGen: An automated system for generating predictive 3D pharmacophore models.. Pharmacophore perception, development, and use in drug design.

[46] Jones G, Willett P, Glen RC, Leach AR, Taylor R (1997). Development and validation of a genetic algorithm for flexible docking.. J Mol Biol.

[47] Shoemaker RH (2006). The NCI60 human tumour cell line anticancer drug screen.. Nat Rev Cancer.

