# Supplementary figures and images for: Exploring DNA Topoisomerase I Ligand Space in Search of Novel Anticancer Agents
Source: PLoS One. 2011 Sep 22;6(9):e25150. doi: 10.1371/journal.pone.0025150 (PMC3178613; doi:10.1371/journal.pone.0025150)

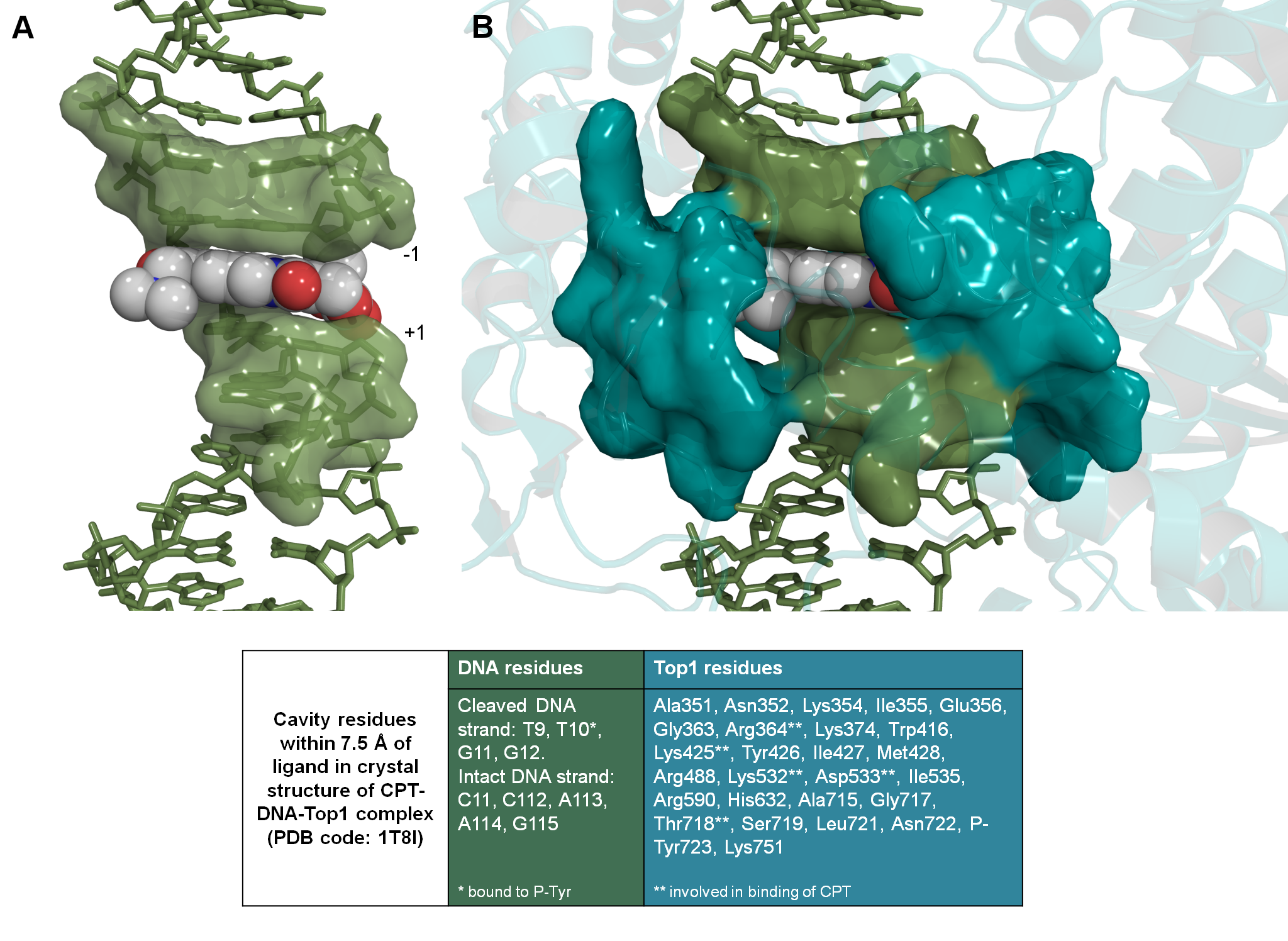

Supplement: Figure S1 — Binding site for the docking simulations. The binding site was defined from the position of the ligand, camptothecin, in the crystal structure ternary complex with a DNA fragment and the top1 enzyme (PDB code 1T8I [23]) and includes both DNA (green) and protein (aqua) residues. (TIF) [file pone.0025150.s001.tif]
