# Supplementary material for: Exploring DNA Topoisomerase I Ligand Space in Search of Novel Anticancer Agents
Source: PLoS One. 2011 Sep 22;6(9):e25150. doi: 10.1371/journal.pone.0025150 (PMC3178613; doi:10.1371/journal.pone.0025150)
Supplement: Table S1 — Training set for ligand-based pharmacophore generation. 2D structures, IC50 values (in µM) for the inhibition of Top1, and references for molecules used in pharmacophore generation are given. (DOCX) [file pone.0025150.s002.docx]

Table S1 Training set for ligand-based pharmacophore generation

| **Structure** | **IC_50_ (µM)** | **Structure** | **IC_50_ (µM)** |
| --- | --- | --- | --- |
|  | 0.011 [30] |  | 0.2 [29] |
|  | 0.015 [32] |  | 0.268 [31] |
|  | 0.015 [30] |  | 0.34 [29] |
|  | 0.028 [31] |  | 0.505 [31] |
|  | 0.033 [31] |  | 0.679 [30] |
|  | 0.038 [32] |  | 1.1 [29] |
|  | 0.071 [31] |  | 1.461 [31] |
|  | 0.086 [32] |  | 1.8 [28] |
|  | 0.1 [29] |  | 100 [28] |
|  | 3.1 [28] |  | 100 [28] |
|  | 4 [28] |  | 100 [28] |
|  | 9.328 [31] |  | 100 [28] |
|  | 12.1 [28] |  | 100 [32] |
|  | 0.16 [32] |  |  |
