# Supplementary material for: Exploring DNA Topoisomerase I Ligand Space in Search of Novel Anticancer Agents
Source: PLoS One. 2011 Sep 22;6(9):e25150. doi: 10.1371/journal.pone.0025150 (PMC3178613; doi:10.1371/journal.pone.0025150)
Supplement: Table S2 — Compounds that did not pass the first docking round. Details on the hit list, the rank, the NSC code are given on compounds failing the first round of docking, as well as reasons for failure. (DOCX) [file pone.0025150.s003.docx]

Table S2 Compounds that did not pass the first docking round

| List | Rank | Compound | Docking result (docking round 1) |
| --- | --- | --- | --- |
| *1* | 6 | NSC 0026808 | - Moderate docking scores - Number of docking clusters high - Although interactions with DNA detected and ligand occupies binding pocket, only few interactions with protein present |
| *1* | 10 | NSC 0289540 | - Number of docking clusters high - Although interactions with DNA detected and ligand occupies binding pocket, no interactions with protein present in the docking poses |
| *1* | 11 | NSC 0609550 | - Moderate docking scores - Number of docking clusters high - Although ligand occupies binding pocket, most of the poses show no intercalation |
| *1* | 13 | NSC 0026809 | - Number of docking clusters high - Although interactions with DNA detected and ligand occupies binding pocket, no interactions with protein present |
| *2* | 9 | NSC 0092903 | - Unknown atom types (As), no docking possible |
| *2* | 14 | NSC 0059242 | - Although ligand occupies binding pocket, many of the poses show no intercalation and few interactions with protein |
